# Polypharmacy and severe potential drug-drug interactions among older adults with cardiovascular disease in the United States

**DOI:** 10.1186/s12877-021-02183-0

**Published:** 2021-04-07

**Authors:** Marwan Sheikh-Taha, Myriam Asmar

**Affiliations:** Department of Pharmacy Practice, Lebanese American University, Byblos, Lebanon

**Keywords:** Polypharmacy, Drug interactions, Cardiovascular disease, Geriatrics

## Abstract

**Background:**

Polypharmacy continues to be a topic of concern among older adults and puts patients at increased risk of potential drug-drug interactions (DDIs) and negative health outcomes. The objective of this study was to assess the prevalence of polypharmacy among older adults with cardiovascular disease (CVD) and to identify severe potential DDIs.

**Methods:**

A retrospective chart review was conducted in a tertiary care center over a three-month period where we reviewed home medications of older adults upon hospital admission. Inclusion criteria were age ≥ 65 years, history of CVD, and admission to the cardiology service. Polypharmacy was defined as 5 or more medications taken concomitantly, hyper-polypharmacy was defined as 10 or more medications taken concomitantly, and severe potential DDIs were considered to be those belonging to category D or X using Lexicomp® Drug Information Handbook. Category D interaction states that modification of therapy should be considered while category X states that the combination should be absolutely avoided.

**Results:**

A total of 404 patients with a mean age of 76.6 ± 7.4 years were included. Patients were taking an average of 11.6 ± 4.5 medications at home and 385 (95%) received polypharmacy, 278 (69%) received hyper-polypharmacy, and 313 (77.5%) had at least one severe potential DDI. Under category D, the most common potential DDIs were drugs with additive central nervous system (CNS) depressant effect and drugs that increase the risk of QT prolongation. Under category X, the most common potential DDIs were non-selective β-blockers that may diminish the bronchodilator effect of β_2_ agonists and drugs with anticholinergic properties that enhance the ulcerogenic effect of oral solid potassium.

**Conclusions:**

Polypharmacy, hyper-polypharmacy, and severe potential DDIs are very common in older adults with CVD. Clinicians should vigilantly review patients’ drug records and adjust therapy accordingly to prevent adverse drug reactions and negative health outcomes.

## Background

The use of multiple medications, known as polypharmacy, is commonly observed among the older adults due to multiple, concurrent and chronic health conditions and continues to be a topic of concern [1, 2]. Polypharmacy is usually defined as taking 5 or more medications concurrently while hyper-polypharmacy is defined as taking 10 or more medications concurrently [3]. The prevalence of polypharmacy varies in different populations and increases with age. In a large study involving 1,742,336 older adults, the prevalence of polypharmacy was 44% [2]. In a Scottish Polypharmacy Guidance, it was reported that up to 11% of unplanned hospital admissions were related to harm from polypharmacy and about 50% of these were preventable [4]. Furthermore, a prospective study of 5052 older adults in Spain reported that polypharmacy increased the risk of mortality by almost 1.8 times [5].

Although the addition of a medication is usually clinically significant and aims at improving the patient’s health, it can put the patient at an increased risk of potential drug-drug interaction (DDI), and drug-disease interactions_._ Clinically significant DDIs manifest as a decline in therapeutic effect of a drug, increased occurrence of adverse drug reactions and compromised treatment outcomes [6]. Severe potential DDIs are those that are life threatening and/or require medical treatment or an intervention to minimize or to prevent the severe adverse effects.

Cardiovascular disease (CVD) is a group of disorders of the heart and blood vessels and includes coronary heart disease, cerebrovascular disease, hypertension, peripheral arterial disease, heart failure, and arrhythmia. According to the American Heart Association, the incidence of CVD in US is around 75% from 60 to 79 years, and 86% in those above the age of 80 [7]. Evidence-based practice for the treatment of CVD recommends a combination of different medications to treat a particular disease [8]_._ Consequently, polypharmacy as well as potential DDIs, and their associated consequences are expected to be observed in older adults with CVD.

The aim of this study is to assess the prevalence of polypharmacy among a sample of older adults with CVD admitted to a tertiary care center in the USA and to analyze the most common and severe potential DDIs occurring in this patient population.

## Methods

This retrospective chart review was conducted at a tertiary center, Huntsville Hospital, Madison County Alabama, USA between March and May 2016. The cohort in this study was also assessed for the use of pain medications [9]. We reviewed the home medications of older adults who were admitted to the cardiology service during the study period. Inclusion criteria were age ≥ 65 years, history of CVD, taking prescription medications at home, and admission to the cardiology ward. Exclusion criteria included patients with missing information in their data when collected or those with moderate to severe cognitive impairment [10].

For the purpose of this study polypharmacy was defined as 5 or more medication taken concomitantly and hyper-polypharmacy was defined as 10 or more medications taken concomitantly [2]_._ Severe potential DDIs were considered to be those belonging to category D or X using Lexicomp Online 2019 Drug Information Handbook [11]. According to Lexicomp®, category A indicates no known interaction, category B indicates drug interaction with little or no clinical data to support it, and category C indicates that two drugs may interact with each other and the benefits of concomitant use of these medications usually overweigh the risks. Category D interaction states that modification of therapy should be considered while category X states that the combination should be absolutely avoided. Lexicomp® is considered one of the best performing potential DDI screening programs [12, 13].

Data were collected from patients’ charts using structured data collection format and analyzed through the SPSS program. Chart abstraction was done by one clinical pharmacist and was reviewed by another in order to reduce error. Data collected included demographic data, condition or disease causing hospital admission, comorbidities, and home medications. Data are presented as mean and standard deviation or median (25–75% interquartile range [IQR]) for continuous variables, and numbers (percentages) for categorical variables. Ethical approval for this study was obtained from Huntsville Hospital’s Institutional Review Committee.

## Results

The medical records of 404 patients that were admitted to the cardiology service and met the inclusion criteria were utilized for this study. Five patients were excluded due to moderate or severe dementia. The mean age of included patients was 76.6 ± 7.4 years and males made up 52.2% (211) of the study population. Patients had a median of 6 comorbidities (IQR 4–8); hypertension was the most common (75.0%), followed by dyslipidemia (52.2%), coronary artery disease (49.5%), and heart failure (41.8%). Table 1 describes patient characteristics and their comorbidities.

**Table 1:** Patient characteristics

	***N*** = 404
**Gender**	
Females	193 (47.8)
Males	211 (52.2)
**Age (years)**	
Mean ± SD	76.6 ± 7.4
**Reason for admission**	
Rule out cardiac etiology	127 (31.4)
Decompensated heart failure	80 (19.8)
Acute coronary syndrome	68 (16.9)
Atrial Fibrillation	43 (10.6)
Syncope	16 (4.0)
Bradycardia	13 (3.2)
**Type of cardiovascular comorbidity**	
Hypertension	303 (75.0)
Dyslipidemia	211 (52.2)
Coronary artery disease	200 (49.5)
Heart failure	169 (41.8)
Atrial fibrillation/flutter	145 (35.9)
Diabetes mellitus	143 (35.4)
Cardiac valve disease	61 (15.1)
Cerebrovascular accident	52 (12.9)
Peripheral vascular disease	47 (11.6)
Deep venous thrombosis / pulmonary embolism	25 (6.2)
**Type of non-cardiovascular comorbidity** (incidence > 10%)	
Chronic kidney disease	115 (28.5)
COPD	84 (20.8)
GERD	79 (19.6)
Hypothyroidism	78 (19.3)
Cancer	77 (19.1)
Benign prostatic hyperplasia	65 (16.1)
Arthritis	63 (15.6)
Anemia	47 (11.6)

Data are mean ± standard deviation or n (%)

*COPD* Chronic obstructive pulmonary disease, *GERD* Gastroesophageal reflux disease

On average, each patient was taking 11.6 ± 4.5 medications at home. A total of 385 (95%) of patients received polypharmacy and 278 (69%) received hyper-polypharmacy. Table 2 describes the number of medications used at home and classes of medications most commonly used. A total of 1080 severe potential DDI’s were identified, 902 (83.5%) fall under category D rating and 178 (16.5%) fall under category X. Overall, 313 (77.5%) patients had at least one severe potential DDI. The most frequent category D DDI’s were drugs with additive central nervous system (CNS) depressant effect (29.5%), followed by drugs that increase the risk of QT prolongation (8.9%), and interactions affecting drug absorption (8.9%). The most common category X DDI’s were non-selective β-blockers that may diminish the bronchodilator effect of β_2_ agonists (24.7%), drugs with anticholinergic properties that enhance the ulcerogenic effect of oral solid potassium dosage forms (24.7%), followed by concomitant use of highest risk QTc-prolonging agents with any other QTc-prolonging agent (14.6%). Table 3 describes the most commonly identified severe potential DDI’s and potential severe adverse effects caused by such interactions.

**Table 2:** Number and classes of home medications

	***N*** = 404
**Number of medications**	
≤ 4	19 (5)
≥ 5	385 (95)
5–9	107 (26)
≥ 10	278 (69)
Total number of medications	4669
Median (IQR)	12 (9–14)
**Classes of medications commonly used**	
Antihypertensive	336 (83.2)
Antiplatelet	280 (69.3)
Dyslipidemia	250 (61.9)
Loop diuretics	161 (39.9)
Antidiabetic	143 (35.4)
Anticoagulant	115 (28.5)
Antidepressant/anxiolytic	110 (27.2)
Proton pump inhibitors	108 (26.8)

Data are Median [P25; P75] or n (%)

**Table 3:** Most common severe potential drug-drug interactions among home medications and potential severe adverse effects

Category D *N* = 902	Potential severe adverse effects^a^	Number of DDIs (%)
Additive CNS depressant effect (CNS depressant* + Opioidanalgesic ***or*** **two CNS depressants)** * Including antipsychotics, benzodiazepines, TCAs, musclerelaxants, sedating antihistamines, and sedative-hypnotics.	Hypotension, sedation, respiratory depression	**266 (29.5)**
**Additive QT prolongation effect** Mostly: **a**miodarone, aripiprazole, citalopram, fluconazole,mirtazapine, and ondansetron.	Torsades de pointes (TdP), death	**80 (8.9)**
**Interactions affecting drug absorption** ➢ Sucralfate + digoxin, warfarin, or furosemide ➢ Levothyroxine **+** minerals, or lanthanum ➢ Quinolones or tetracyclines + minerals ➢ Bile acid sequestrants + hydrochlorothiazide or statins	Variations in systemic drug availability and henceclinical efficacy	**80 (8.9)**
**Antiplatelet agents + oral anticoagulant**	Increased risk of bleeding, both major and minor	**65 (7.2)**
**Statins + drugs that increase their levels** (Simvastatin + amiodarone, amlodipine, diltiazem, or ranolazine) (Atorvastatin + diltiazem or verapamil)	Increased risk of muscle toxicity, rhabdomyolysis	**56 (6.2)**
**Drug combination that can cause bradycardia/AV block** Mostly: β-blockers, clonidine, diltiazem verapamil, and centralα2agonists.	Bradycardia, AV block	**36 (4)**
**Esomeprazole/omeprazole + Clopidogrel**	Increase in incidence of major adverse cardiac events	**35 (3.9)**
**Aspirin + NSAID**	Increase in incidence of major adverse cardiac events	**25 (2.8)**
**SSRI + SSRI/SNRI/TCA**	Increased risk of serotonin syndrome/serotonin toxicity	**25 (2.8)**
**NSAID + SSRI**	Increased risk of bleeding	**13 (1.4)**
**NSAIDs + loop diuretics**	Reduced diuretic effect, acute kidney injury	**12 (1.3)**
**ACEI + ARB**	Hyperkalemia, acute kidney injury	**11 (1.2)**
**Oral anticoagulant + estradiol**	Increased risk of thromboembolism	**8 (0.9)**
**Colchicine + statins**	Increased risk of muscle toxicity, rhabdomyolysis	**7 (0.8)**
**Category X** ***N* = 178**		
**Nonselective β-blocker** (carvidolol, propranolol) **+ β**_**2**_ **agonist**(albuterol, formoterol)	Bronchospasm, could be severe	**44 (24.7)**
**Anticholinergic agents**^**#**^ **+ oral solid potassium dosage forms** ^#^ Amitriptyline, benztropine, cyproheptadine, diphenhydramine,olanzapine, oxybutynin, promethazine, quetiapine, or solifenacin.	Increased risk of ulcerative/stenotic lesions	**44 (24.7)**
**Concomitant use of highest risk QTc-prolonging agents**^**&**^ **with any other QTc-prolonging agent** ^&^ Amiodarone, citalopram, sotalol, dronedarone, quetiapine,ziprasidone	Torsades de pointes (TdP), death	**26 (14.6)**
**Dual anticholinergic agents**	Confusion, dry mouth, blurred vision, arrhythmia, falls	**24 (13.5)**
**Sucralfate + Vitamin D analogs**	Increased risk for aluminum accumulation/toxicity	**10 (5.6)**
**Concomitant vitamin D analogs**	Vitamin D toxicity, hypercalcemia	**6 (3.4)**
**Rivastigmine + β-blocker**	Bradycardia, syncope	**4 (2.2)**
**Cyclosporine + atorvastatin**	Myopathy, rhabdomyolysis	**2 (1.1)**

*ACEI* Angiotensin-converting-enzyme inhibitor, *ARB* Angiotensin II receptor blocker, *CNS* Central nervous system, *NSAID* Non-steroidal anti-inflammatory drug, *SSRI* Selective serotonin reuptake inhibitor, *SNRI* Serotonin norepinephrine reuptake inhibitor, *TCA* Tricyclic antidepressant

^a^ Reference: Lexicomp®

## Discussion

Among the geriatric population with multiple comorbidities, polypharmacy is a common phenomenon. Polypharmacy carries a high risk of DDIs and continues to be a matter of concern since its consequences may vary from minor health hazard to fatality [2, 5]. This study was done in the aim of describing the prevalence of polypharmacy and the type of severe potential DDI’s among older adults with CVD.

In general, the prevalence of polypharmacy varies widely according to the age group, definition used, healthcare and geographical setting of the study [2]. In the United States, the prevalence of polypharmacy was 26% of all adults, and 61% of adults over 65 years of age had two or more chronic condition [14]. In Sweden and Korea, the prevalence of polypharmacy among older adults was 44.0 and 86.4% respectively [15, 16]. In our study, the prevalence of polypharmacy was 95% and hyper-polypharmacy was 65%. The number of comorbidities, older age, and CVD were significantly linked with occurrence of polypharmacy and hyper-polypharmacy in various studies which could explain the higher numbers seen in our study where we studied older adults with CVD who had a mean of 6 comorbidities [17–19]. It should not be assumed that polypharmacy is poor care and it should be interpreted in the clinical context for individual patients. Clinicians should distinguish between appropriate and inappropriate polypharmacy and reduce inappropriate polypharmacy and severe potential DDIs. The use of relevant indicators could help in identifying the appropriateness of polypharmacy as suggested by the Scottish Polypharmacy Guidance and Burt and colleagues [4, 20]. Nevertheless, more studies are needed to confirm their usefulness and feasibility. In addition, while a DDI screening program may classify the concomitant administration of antiplatelets and anticoagulants as DDIs under category D due to increased risk of bleeding, this drug combination can be appropriate in patients suffering from ischemic heart disease and atrial fibrillation. Hence, DDI screening programs cannot replace good clinical judgment.

Around three-fourth of the included patients had at least one severe potential DDI. In an Italian study involving older adults, only 16% of studied patients had at least one potentially severe DDI and the cardiovascular drugs were the most frequently involved. However, severe potential DDIs were defined using the Italian interaction database [21]. The higher numbers seen in our study could be explained by including patients with CVD and by using Lexicomp® to define severe potential DDIs.

In the current study, the most commonly observed severe potential DDIs under category D (modification of therapy should be considered) were drugs with additive CNS depressant effect, followed by drugs that increase the risk of QT prolongation, and interactions affecting drug absorption.

### Drugs with additive CNS depressant effect

Our study analyzed two types of interactions under this category; the combination of opioid analgesic with drugs that possess CNS depressant effect or dual agents with CNS depressant effect. It is recommended to avoid the concomitant use of CNS depressant agents unless alternative treatment is not possible. When combined, the clinician should prescribe the lowest possible dose and duration of each drug while achieving the desired clinical effect [22]. It is also crucial to monitor patients for any sign and symptoms of CNS depression including hypotension, sedation, and respiratory difficulties.

### Additive QT prolongation effect

An extensive list of medication such as azole antifungal, antiarrhythmic, antiemetic, antipsychotic and antidepressant drugs can prolong the QTc interval, and the concurrent use of these medications should be avoided if possible. Older adults, female gender, heart disease, bradycardia, congenital long QT syndrome, electrolyte disturbances (hypokalemia, hypomagnesemia, and hypocalcemia), diuretic treatment and patients with hepatic drug metabolism impairment are at a higher risk than the general population to suffer from this life-threatening side effect [23]_._ Considering the wide range of clinically essential drugs that have QT prolongation properties, there should be a need to implement protocols to emphasize close electrocardiogram (ECG) monitoring when concomitant administration of such medications is necessary.

### Interactions affecting drug absorption

Sucralfate and bile acid sequestrants can bind to other drugs in the gastro-intestinal (GI) tract, reducing their absorption when administered simultaneously. In addition, the absorption of oral quinolones, tetracyclines, and levothyroxine can be reduced when co-administered with minerals (such as iron, potassium, zinc, and magnesium). To avoid this potential DDI, it is recommended that interacting drugs be spaced several hours apart from other drugs [24]. The potential clinical implications of this potential DDI must be taken into account in order to minimize variations in systemic drug availability and hence in clinical efficacy.

The most commonly observed severe potential DDIs under category X (the combination should be avoided) were β_2_ agonists and nonselective β-blockers, and anticholinergic agents and oral solid potassium.

### β_2_ agonists and nonselective β-blockers

Nonselective β-blockers that act on β_1_ and β_2_ receptors will antagonize the effect of β_2_ agonists. The following interaction is especially important in asthmatic and chronic obstructive pulmonary disease (COPD) patients that need the bronchodilatory effects of β_2_ agonists as part of their treatment to avoid severe bronchospasm. In patients with respiratory conditions, selective β_1_ blockers are not associated with a significant increase in exacerbations and thus should be prescribed and preferred over nonselective agents when there is a compelling indication such as post myocardial infarction or heart failure with reduced ejection fraction [25]_._

### Anticholinergic agents and oral solid potassium dosage forms

The combination of oral solid potassium dosage forms and drugs with anticholinergic properties may delay solid potassium passage through the GI tract, which can increase the local exposure to high potassium concentration and consequently, increase the risk of ulcerative/stenotic lesions. Agents with greater anticholinergic effects are likely of more concern than those with lesser anticholinergic effects, and liquid or effervescent potassium preparations seem to be safer alternatives [11].

Our study had some limitations of its own. A major limitation is that the study was limited to describing potential DDIs on admission to a cardiology service, and that other important aspects were not assessed. These aspects include assessing the clinical relevance of potential DDIs at individual level, analyzing how whether and how these DDIs were handled during hospital admission, and analyzing the factors associated with these potential severe DDIs. In addition, the study was a retrospective chart review and data was collected from a single medical center. A multi-centered study would have tackled probable differences in prescribing patterns and would have allowed the data to be more generalizable. In addition, due to the nature of the study some data was missing, and different forms of bias might have been introduced. Furthermore, in our study we did not assess whether the polypharmacy was appropriate or inappropriate.

## Conclusion

In this study, polypharmacy, hyper-polypharmacy, and severe potential DDIs were very common in older adults with CVD. Healthcare professionals should carefully review every patient’s drug record upon each visit, identify unnecessary medications and severe potential DDIs that are potentially harmful, and adjust therapy accordingly in order to optimize patient care.

## Data Availability

The datasets used and/or analyzed during the current study are available from the corresponding author on reasonable request.

## Abbreviations

CNS: Central nervous system
COPD: Chronic obstructive pulmonary disease
CVD: Cardiovascular diseases
DDI: Drug-drug interaction
ECG: Electrocardiogram
GI: Gastro-intestinal
TdP: Torsades de pointes
